# Development and Characterization of Terbinafine-Loaded Nanoemulgel for Effective Management of Dermatophytosis

**DOI:** 10.3390/gels9110894

**Published:** 2023-11-12

**Authors:** Mayank Phagna, Reena Badhwar, Manvi Singh, Abdulsalam Alhalmi, Rahmuddin Khan, Omar M. Noman, Ahmad Alahdab

**Affiliations:** 1 Department of Pharmaceutics, SGT College of Pharmacy, SGT University, Gurugram 122001, India; mayankphagna12@gmail.com (M.P.); reena_fphs@sgtuniversity.org (R.B.); 2 Department of Pharmaceutics, School of Pharmaceutical Education and Research, Jamia Hamdard, New Delhi 110062, India; asalamahmed5@gmail.com (A.A.); rkm.hamdard@gmail.com (R.K.); 3 Department of Pharmacognosy, College of Pharmacy, King Saud University, P.O. Box 2457, Riyadh 11451, Saudi Arabia; 4 Institute of Pharmacy, Clinical Pharmacy, University of Greifswald, Friedrich-Ludwig-Jahn-Str. 17, 17489 Greifswald, Germany

**Keywords:** terbinafine hydrochloride, dermatophytosis, nanoemulgel, nanoemulsion, texture analysis

## Abstract

Dermatophytosis, the most prevalent fungal infection, is witnessing a rising incidence annually. To address this challenge, we developed a terbinafine-loaded oil-in-water nanoemulsion (TH-NE) through the aqueous microtitration method. The formulation comprised olive oil (oil phase), Span 80 (surfactant), and propylene glycol (co-surfactant). Pseudo-phase ternary diagrams and thermodynamic studies underscored the stability of TH-NE. Employing the Box–Behnken design (BBD), we optimized TH-NE, which resulted in a remarkable particle size of 28.07 nm ± 0.5, a low polydispersity index (PDI) of 0.1922 ± 0.1, and a substantial negative zeta potential of −41.87 mV ± 1. Subsequently, TH-NE was integrated into a 1.5% carbopol matrix, yielding a nanoemulgel (TH-NEG). Texture analysis of TH-NEG demonstrated a firmness of 168.00 g, a consistency of 229.81 g/s, negative cohesiveness (−83.36 g), and a work of cohesion at −107.02 g/s. In vitro drug release studies revealed an initial burst effect followed by sustained release, with TH-NEG achieving an impressive 88% release over 48 h, outperforming TH-NE (74%) and the marketed formulation (66%). Ex vivo release studies mirrored these results, with TH-NEG (86%) and TH-NE (71%) showcasing sustained drug release in comparison to the marketed formulation (67%). Confocal microscopy illustrated that TH-NEG and TH-NE penetrated to depths of 30 µm and 25 µm, respectively, into the epidermal layer. Furthermore, dermatokinetic studies highlighted the enhanced drug penetration of TH-NEG compared to TH-NE through mouse skin. In summary, our study establishes TH-NEG as a promising carrier for terbinafine in treating dermatophytosis, offering improved drug delivery and sustained release potential.

## 1. Introduction

Dermatophytosis, which represents approximately 25% of all skin mycoses globally, is a prevalent form of infectious disease worldwide [1]. This condition results from superficial fungal infections caused by three categories of keratinophilic fungi: *Trichophyton* (affecting skin, nails, and hair), *Microsporum* (infecting skin and hair), and *Epidermophyton* (impacting the skin and nails) [2]. The prevalence of dermatophytosis is notably high in tropical and subtropical regions such as India due to the hot and humid climate. According to a recent study, tinea corporis emerged as the most frequently encountered infection, affecting 65.45% of the patients surveyed [3]. Various topical antifungal medications are accessible in the market to address dermatophytosis. However, resistance has developed against commonly used topical antifungal drugs for this condition, including clotrimazole, econazole, ketoconazole, and miconazole. Additionally, these medications may lead to adverse effects such as skin sensitivity and urticaria [4].

Terbinafine has emerged as a promising option for dermatophytosis treatment. It works by blocking the synthesis of ergosterol, a crucial component in fungal cell membranes, through inhibiting squalene epoxidase, an enzyme involved in the fungal membrane synthesis process. Additionally, it hinders the conversion of squalene into lanosterol, inhibiting ergosterol and ultimately causing fungal cell breakdown [5]. Terbinafine HCl cream and powder are employed to treat surface skin infections like jock itch (tinea crusis), athlete’s foot (tinea pedis), and ringworm. This medication has a strong affinity for fat and can accumulate in the skin, nails, and fatty tissues. It undergoes a first-pass effect in the body, resulting in limited oral effectiveness. Moreover, terbinafine is experiencing growing resistance, necessitating the exploration of repurposing options [6].

Delivering medications topically for dermatophytosis offers several advantages. It circumvents the initial metabolism, which, in turn, lowers the systemic drug burden, resulting in fewer systemic side effects. Additionally, it eliminates the potential risks and inconveniences associated with intravenous therapy. Furthermore, topical drug delivery improves treatment effectiveness by mitigating gastrointestinal irritation, inconsistent drug absorption with oral treatments, drug–food interactions, and enzymatic activity [7]. Reducing the treatment expense is achieved through user-friendliness, eliminating the need for trained medical personnel for medication administration. Patient adherence improves when self-administration is straightforward and nonintrusive. Moreover, in case of toxicity, therapy can be promptly halted at any time [8]. Nanoemulgel has emerged as one of the most interesting topical delivery systems as it has a release control system, i.e., hydrogel and nanoemulsion [9]. The nanosized (10 to 100 nm) structure of nanoemulgel enables swift and efficient delivery of active ingredients, ensuring they reach deeper layers of the skin more rapidly. The gelling agent plays a crucial role in enhancing the stability of the nanoemulsion by reducing surface and interfacial tension while also increasing the viscosity of the aqueous phase, thereby improving the topical administration of the drug [10]. Using nanoemulgel enhances drug adhesion to the skin surface and increases its solubility, resulting in a more significant concentration gradient toward the skin. This, in turn, improves drug penetration into the skin and prolongs its retention on the skin [11].

Numerous researchers have concentrated their efforts on creating terbinafine formulations, but they have faced certain constraints, as elaborated below. In their work, Hajare and colleagues crafted an ethosomal gel containing terbinafine hydrochloride to improve transdermal delivery systems. The perfected formula resulted in vesicles of approximately 127.39 ± 2.71 nm in size, a zeta potential of −40.63 ± 2.77 mV, and an entrapment efficiency of 87.55 ± 0.47%. However, it should be noted that skin irritation may arise due to the alcoholic content present in these ethosomes [12]. Nanosponges of butenafine were fabricated with butenafine (100 mg), ethyl cellulose (200 mg), and 0.3% polyvinyl alcohol. It showed a particle size (543 ± 0.67 nm), PDI (0.330 ± 0.02), zeta potential (−33.8 ± 0.89 mV), % entrapment efficiency (71.3 ± 0.34%), and % drug loading (22.8 ± 0.67%), respectively. The drug-loading capacity of nanosponges for the treatment of fungal skin infections was found to be significantly less, which posed difficulty in drug diffusion [13]**.** A terbinafine polymeric nanosponge hydrogel was formulated, demonstrating a flux rate of approximately 0.594 ± 0.22 µg/cm^2^/h and a permeability coefficient of around 0.059 ± 0.022 cm/s. When applied topically, this hydrogel accumulates in the skin and nails, potentially leading to adverse effects such as rashes, irritation, and allergic reactions [14]**.** To address the challenges related to size, toxicity, confinement, and dispersion, our proposal involves creating a nanoemulgel containing terbinafine. This nanoemulgel will feature reduced particle size, a greater drug content, and enhanced permeability, aiming to improve the treatment of dermatophytosis.

## 2. Results and Discussion

### 2.1. Selection of Excipients

Conducting studies on the solubility of drugs is of paramount importance in developing nanoemulsions, as they significantly impact the stability and effective delivery of the formulation. Additionally, it is essential to carefully choose appropriate additives to create a transparent, uniform mixture with a strong ability to bind with the active pharmaceutical ingredient (API).

#### 2.1.1. Screening of Oils 

**Figure 1:**
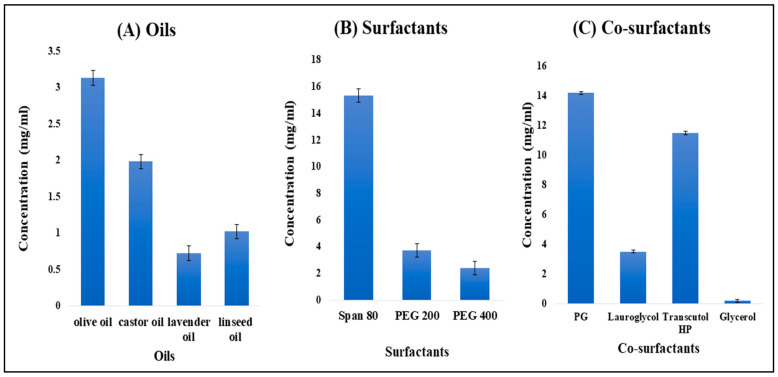
Solubility of TH in different oils (**A**), different surfactants (**B**), and different co-surfactants (**C**).

The researchers conducted a study to explore the solubility of TH in different excipients, and the results are depicted in Figure 1A. Among the various experimental oils tested, the highest solubility of TH (3.13 ± 0.20 mg/mL) was observed in olive oil. Notably, olive oil also possesses germicidal properties, which enhance its suitability as an oily phase for formulation and contribute to a synergistic effect.

#### 2.1.2. Screening of Surfactants

Span 80 was selected as the surfactant because TH showed a maximum drug solubility of 15.31 ± 01.58 mg/mL, as shown in Figure 1B. The solution was found to be clear and transparent. Span 80 is used as a dispersing agent and also provides stability to the nanoemulsion.

#### 2.1.3. Screening of Co-Surfactants

Propylene glycol (PG) was selected as a co-surfactant, as it showed maximum drug solubility of 14.2 ± 0.64 mg/mL, as shown in Figure 1C. It is used as a humectant, solvent, and emollient in the formulation and also helps maintain moisture levels in gel preparations.

### 2.2. Pseudo-Phase Ternary Diagrams

**Figure 2:**
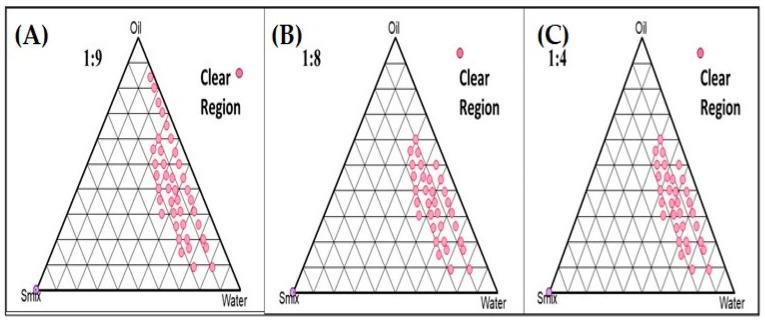
Pseudoternary phase diagrams system containing olive oil (oil phase), Span 80 (surfactant), and propylene glycol (co-surfactant). Dotted area shows O/W NE region in different ratio of surfactant to cosurfactant. (**A**) Smix (1:9); and (**B**) Smix (1:8); and (**C**) Smix (1:4).

A combination of Span 80 and propylene glycol (S_mix_) in the ratio of 1:2 was selected, and the experiment was carried out for different ratios such as 1:9, 1:8, and 1:4 using pseudo-ternary phase diagrams to formulate a clear and transparent NE using the aqueous microtitration dilution method as shown in Figure 2.

### 2.3. Box–Behnken Design (BBD) Mathematical Model Fitting and Optimization of TH-NE

In the current setup, we chose specific independent variables, namely the concentration of oil (µL), surfactant concentration (mL), stirring speed (rpm), and dependent variables, which included particle size (nm), polydispersity index (PDI), and zeta potential (mV). The recorded results are presented in Table 1. We employed the Box–Behnken design (BBD) optimization approach using Design Expert 11 software (developed by Stat-ease Inc., Minneapolis, MN, USA) for statistical analysis, as detailed in Table 2.

#### 2.3.1. Effects of Independent Variables on Particle Size

**Figure 3:**
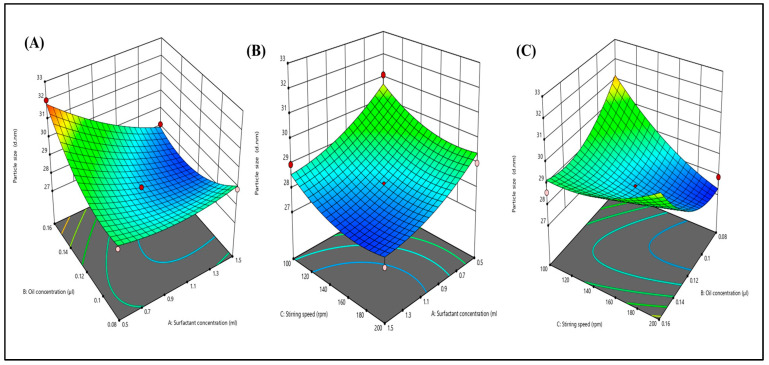
Three-dimensional response surface depicting the interaction effect of the independent variables like oil concentration, surfactant concentration, and stirring speed on particle size (**A**–**C**).

The nanoemulsion size range is from 25.0 nm to 35.0 nm. To determine the particle size distribution, the dynamic light scattering method was used (Figure 3A–C). As stirring speed and surfactant concentration increase, particle size was found to be reduced due to shear and cavitation forces. Both variables were found to be quadratic. It was reported that due to high stirring speed, the particle size reduces, whereas an increase in the concentration of oil increases the particle size. When the particle size is small, it provides a larger surface area that affects the permeation, delivery of the drug, and bioavailability. However, due to the different types of variables present in o/w NE, the particle size increases with an increase in the oil concentration. This can be attributed to the fact that the presence of oil increases the chain length and causes the formation of a larger droplet size.

#### 2.3.2. Effects of Independent Variables on PDI

**Figure 4:**
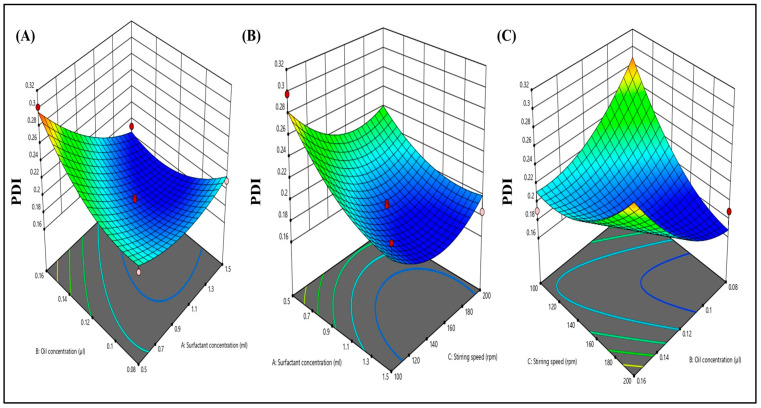
Three-dimensional response surface depicting the interaction effect of the independent variables like oil concentration, surfactant concentration, and stirring speed on PDI (**A**–**C**).

Dynamic light scattering was employed to analyze the PDI distribution. The influence of various factors on PDI is illustrated in 3D response graphs (see Figure 4A–C). The particle size distribution serves as an indicator of both uniformity and the extent of agglomeration within the formulation. Moreover, all 15 formulations exhibited PDI values falling within the range of 0.182 to 0.302, which was deemed appropriate. This is important because a PDI exceeding 0.3 typically indicates particle agglomeration, which could lead to an increase in particle size**.**

#### 2.3.3. Effects of Independent Variables on Zeta Potential

**Figure 5:**
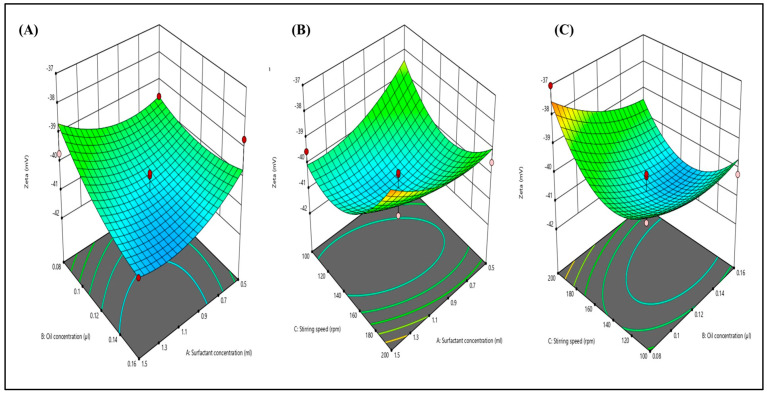
Three-dimensional response surface depicting the interaction effect of the independent variables like oil concentration, surfactant concentration, and stirring speed on zeta potential (**A**–**C**).

The zeta potential was determined using the dynamic light scattering methodology. Figure 5A–C illustrate the impact of various independent factors on the zeta potential. Additionally, all 15 formulations exhibited zeta potential values ranging from −37.02 to −41.87. Consequently, the calculated zeta potential was −41.87 ± 1 mV, signifying the robust stability of the nanoemulsion. The 3D graphs depict that an increase in surfactant concentration and stirring speed leads to a decrease in the zeta potential value. This phenomenon arises from the more substantial negative zeta potential, which results in increased repulsive forces between particles, preventing particle agglomeration.

### 2.4. Particle Size, Polydispersity Index (PDI), and Zeta Potential

**Figure 6:**
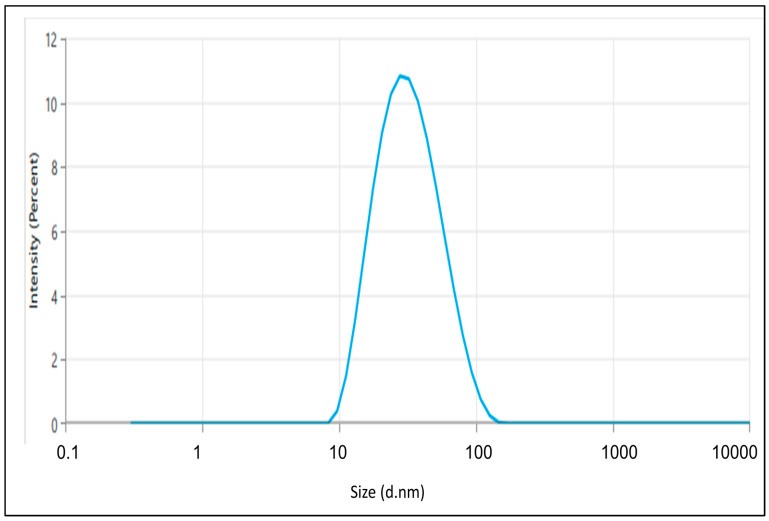
The particle size of the optimized TH-NE.

**Figure 7:**
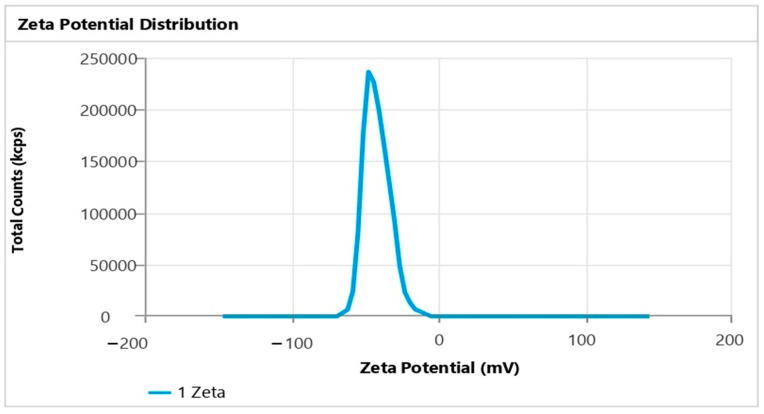
Zeta potential of TH-NE.

The enhanced TH-NE demonstrated a droplet/particle size measuring 28.07 ± 1.43 nm, a PDI of 0.1922 ± 0.014, and a zeta potential of −41.87 ± 0.14 mV, as illustrated in Figure 6 and Figure 7. Having a smaller particle size and uniform particle size distribution facilitate the smooth passage of drugs through membranes, as referenced in [15]. Furthermore, the elevated zeta potential contributes to the stability of the nanoemulsion.

### 2.5. Differential Scanning Calorimetry (DSC)

**Figure 8:**
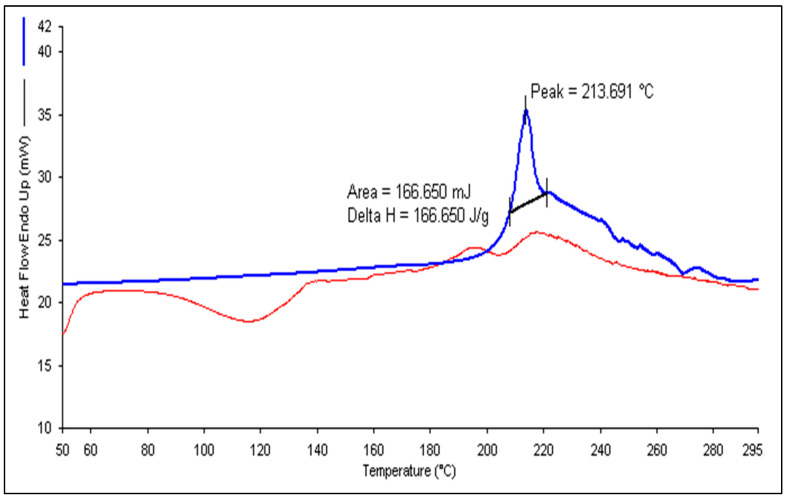
DSC thermogram of pure terbinafine HCl (**A**) and TH-NE (**B**).

The peak observed in Figure 8A indicates that TH undergoes a phase transition at approximately 213.691 °C. This value closely matches the previously reported range of 210 to 214 °C [16]. The DSC analysis revealed a clear and defined peak with endothermic properties, confirming the crystalline nature of the drug. Conversely, when examining the DSC thermogram of the lyophilized and optimized TH-NE formulation, there was no distinct peak corresponding to the drug, as indicated by the peak in Figure 8B. This lack of a sharp peak suggests no drug leakage from the formulation.

### 2.6. Fluorescence Microscopy

**Figure 9:**
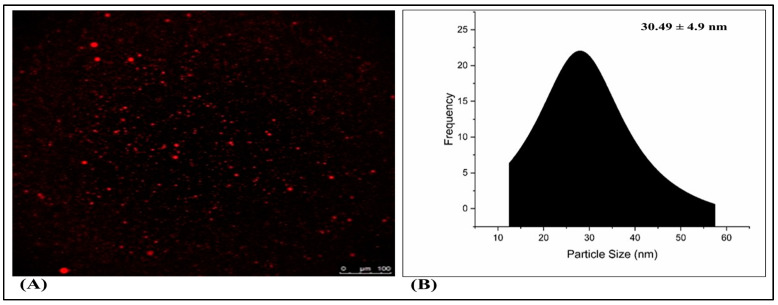
Fluorescence microscopy of TH-NE (**A**,**B**).

A fluorescence microscopy analysis, illustrated in Figure 9A, was employed to examine the surface characteristics of TH-NE. Figure 7B demonstrates that the optimized TH-NE exhibited particle sizes within the range of 30 to 40 nanometers.

### 2.7. Preparation of Drug-Loaded Nanoemulgel Using Optimized Terbinafine HCl Nanoemulsion (TH-NEG)

Several placebo gel formulations containing varying amounts of Carbopol 934^®^ (1%, 1.35%, and 1.5%) underwent assessment, and the 1.5% concentration yielded satisfactory outcomes. Then, 5 mL of the optimized TH-NE was incorporated into the 1.5% carbopol gel, resulting in a well-prepared TH-NEG that exhibited a smooth and uniform texture without coarse characteristics.

### 2.8. Rheological Behavior Studies 

**Figure 10:**
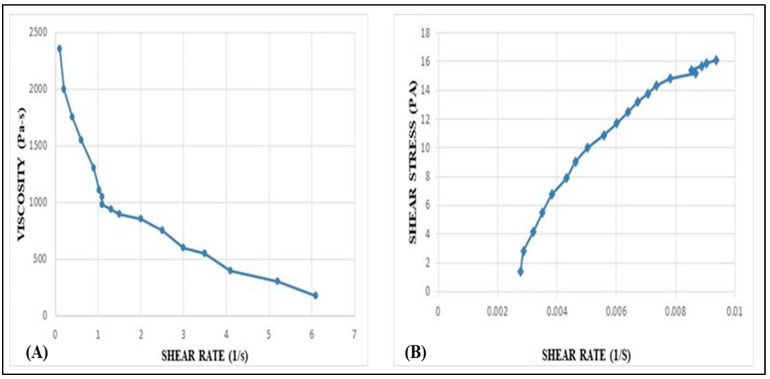
Variation of viscosity vs. shear rate of developed TH-NHG (**A**). Shear stress vs. shear rate of the developed TH-NEG (**B**). Variation of shear stress (τ) and viscosity (η) of developed gel with shear rate (γ).

The formulated TH-NEG exhibited a viscosity of 41.5 Pa·s. Figure 10 illustrates how the viscosity and shear stress of TH-NEG change with varying applied shear rates. TH-NEG displayed non-Newtonian behavior—specifically, shear-thinning pseudoplastic characteristics. This is evident from the fact that its viscosities decreased as the shear rate increased, ranging from 0.1 s^−1^ to 100 s^−1^. These lower viscosities at higher shear rates offer a practical advantage in industrial applications, enabling more efficient flow behavior with reduced energy consumption.

### 2.9. Spreadability and Extrudability 

Spreadability and extrudability are significant in determining how easily it can be applied at the intended location and how efficiently it can be dispensed from the packaging [17]. Therefore, these factors are crucial for ensuring a high level of patient compliance. The optimized formulation demonstrated a spreadability of 14.38 ± 1.55 g.cm/s and an extrudability of 5.78 ± 1.03 g, indicating its suitability for these purposes.

### 2.10. Texture Analysis of Formulation 

**Figure 11:**
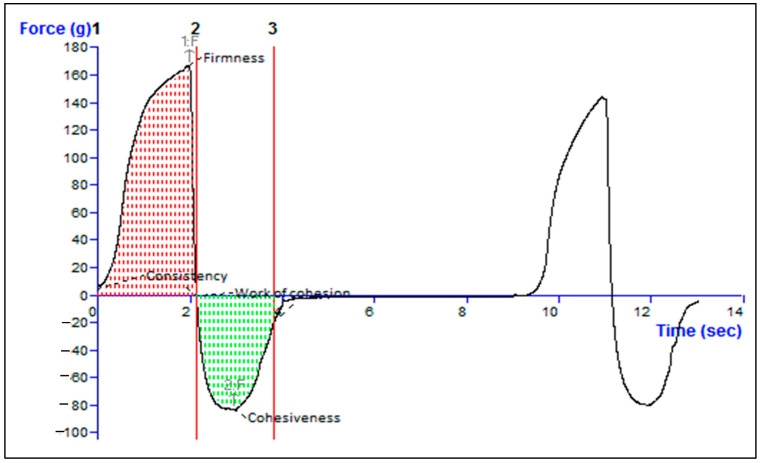
Texture analysis data of the TH-NEG.

The optimized formulation demonstrated strong cohesion with a measurement of −83.36 g, indicating its capacity to adhere to surfaces when subjected to tensile strength. To evaluate the structural integrity of the TH-NEG when subjected to compressive force, its firmness was determined to be 168.25 g, as detailed in Table 3. Various parameters, including consistency, firmness, work of cohesion, and cohesiveness, can be derived from the force–time plot depicted in Figure 11. A higher positive force corresponds to greater gel hardness or firmness, while the negative area beneath the force–time curve characterizes the gel’s cohesiveness.

### 2.11. Homogeneity and pH

The optimized TH-NEG showed sufficient and acceptable homogeneity with no grittiness. The optimized gel had a pH of 5.6 ± 0.12, indicating its skin-friendliness and safety. 

### 2.12. In Vitro Drug Release Study

**Figure 12:**
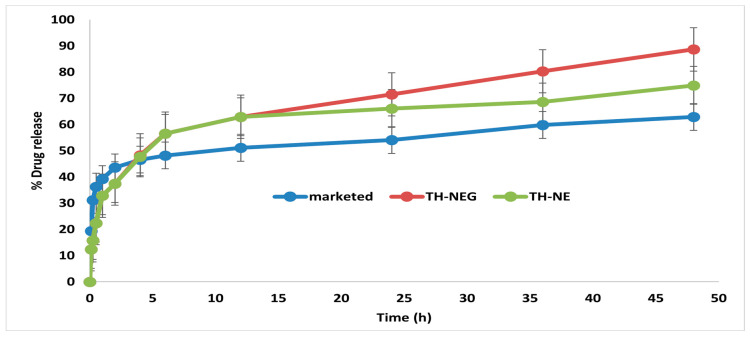
In vitro release of different formulation.

The drug release characteristics of the optimized TH-NE, TH-NEG, and the commercially available formulation were investigated using a dialysis membrane and graphically represented in Figure 12. The percentage of drug released after 48 h was determined to be 88.64% ± 4.15% for TH-NEG, 62.86% ± 3.62% for the marketed formulation, and 74.94% ± 3.22% for TH-NE.

### 2.13. Ex Vivo Drug Release Study

**Figure 13:**
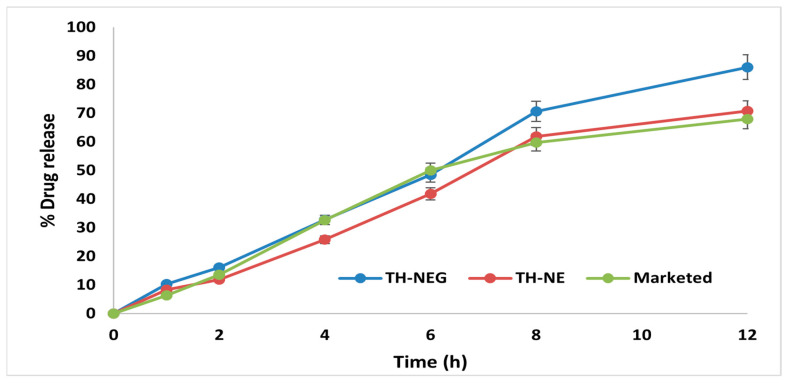
Ex vivo release of different formulation.

Ex vivo drug release experiments were conducted using freshly excised mouse skin, with hair removal prior to sacrificing the mice. The percentage of drug release for three formulations, namely TH-NEG, TH-NE, and a commercially available product, was assessed within a Franz diffusion cell. The outcomes were computed and are visually represented in Figure 13. After 12 h, the drug release percentages were determined to be 86.03% ± 4.15% for TH-NEG, 62.86% ± 1.88% for the marketed formulation, and 70.71% ± 2.22% for TH-NE. 

### 2.14. Confocal Laser Scanning Microscopy (CLSM)

**Figure 14:**
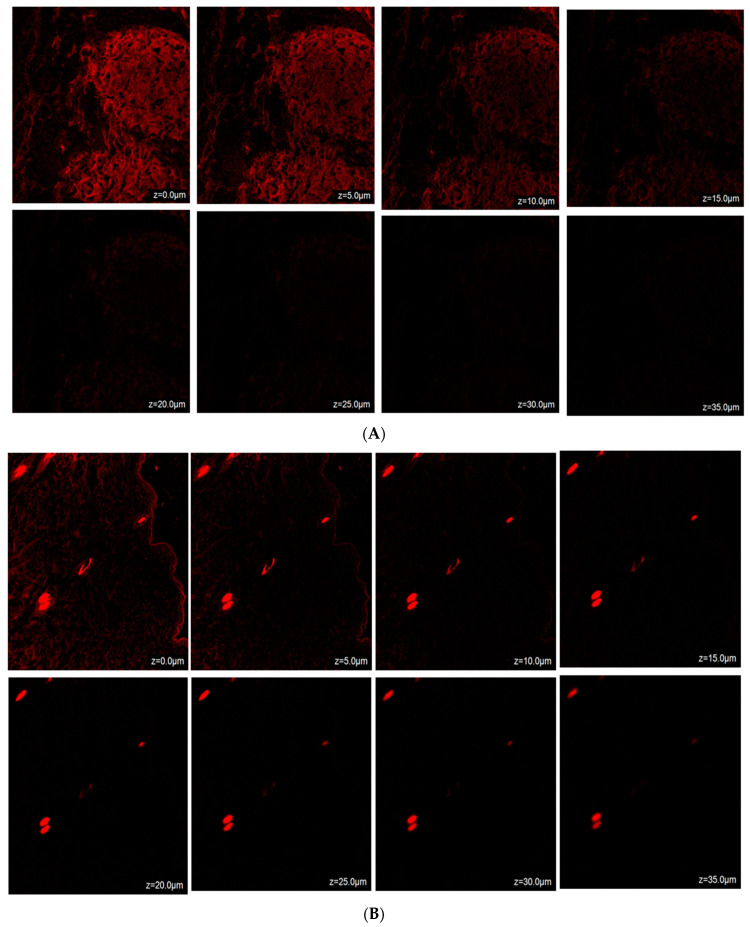
(**A**) Confocal laser scanning microscopy for TH-NEG. (**B**) Confocal laser scanning microscopy for TH-NE.

Confocal microscopic imaging was employed to conduct skin permeation experiments. Following ex vivo studies on mouse skin samples, the results from confocal laser scanning microscopy (CLSM) indicated that TH-NE and TH-NEG permeated the stratum corneum layer of the epidermis. When TH-NE and TH-NEG were exposed to a fluorescent dye (rhodamine B), they were observed to penetrate to depths of 25 μm for TH-NE (as depicted in Figure 14A) and 30 μm for TH-NEG (as depicted in Figure 14B).

### 2.15. Dermatokinetic Study

**Figure 15:**
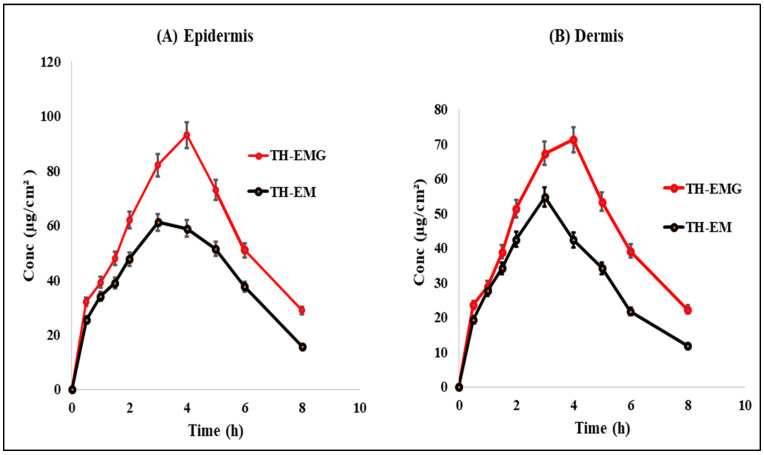
Dermatokinetic profile of TH in (**A**) epidermis and (**B**) dermis.

Figure 15A,B illustrate the dermatokinetic profiles of the TH-NEG and TH-NE formulations. The TH-NEG demonstrated a maximum concentration (C_maxskin_) of 93.03 µg/cm^2^ and 71.23 µg/cm^2^ in the epidermis and the dermis, respectively, which were significantly higher than that from TH-NE (C_maxskin_ values of 61.08 µg/cm^2^ in the epidermis and 54.65 µg/cm^2^ in the dermis) (*p* < 0.05). The time taken to reach the peak concentration of the drug (T_max_) was 4 h for both the epidermis and dermis in the case of TH-NEG, while for TH-NE, it was 3 h for both skin layers. The area under the curve from 0 to 8 h (AUC_0–8h_) for TH-NEG in the epidermis was 459.315, and in the dermis, it was 357.04. In contrast, the epidermis and dermis displayed AUC_0–8h_ values of 328.20 and 248.05, respectively, for TH-NE. Furthermore, the elimination rate constant (Ke) for TH-NEG was 0.825 in the epidermis and 0.822 in the dermis. In contrast, TH-NE exhibited Ke values of 0.893 in the epidermis and 0.866 in the dermis. The C_Skin-max_ of TH in the emulgel was significantly higher (*p* < 0.05) compared to the C_Skin-max_ in the emulsion, as mentioned earlier, in both the epidermal and dermal layers. Similarly, the AUC_0–8h_ (area under the curve from 0 to 8 h) of TH in the emulgel was significantly higher (*p* < 0.05) than the AUC_0–8 h_ in the emulsion, as observed in both the dermal and epidermal layers. In summary, the higher concentration of TH achieved in the skin layers with TH-NEG resulted in greater availability of the drug in both the epidermal and dermal layers, thereby enhancing drug targeting to the surface layer of the skin.

## 3. Conclusions

In this study, terbinafine nanoemulgel was fabricated for the treatment of dermatophytosis. The strategy was to develop an effective formulation through the preparation of the nanoemulsion with a small size and uniform particle size distribution. Optimized nanoemulsion was then incorporated into a gel base, which enhanced drug delivery through the layers of the skin. Nanoemulgel exhibited controlled and sustained drug release, permeation, and diffusion through the layers of the skin. Furthermore, it was concluded that there is an enhanced bioavailability of the drug in different layers of the skin. This formulation allows for better drug penetration into the skin, potentially leading to improved therapeutic outcomes and patient compliance. Further research and clinical studies are needed to fully evaluate the effectiveness and safety of terbinafine nanoemulgel for treating dermatophytosis.

## 4. Materials and Methods

### 4.1. Materials

Terbinafine HCL was procured from Saptagir Laboratories Pvt. Ltd., Telengana, India. Olive oil, propylene glycol, Span 80, Carbopol 934, methanol, and acetonitrile, which were all of analytical grade, were purchased from Molychem, Mumbai, India.

### 4.2. Methodology 

#### 4.2.1. Selection of Excipients

The key to formulating and ensuring the stability of a nanoemulsion lies in the thorough evaluation of suitable oils, surfactants, and co-surfactants. Consequently, various oils, including olive, castor, lavender, and linseed oils, were assessed for this formulation based on their compatibility with the drug and solubility. The surfactants and co-surfactants chosen for assessment included Span 80, PEG 200, PEG 400, propylene glycol (PG), Lauroglycol, Transcutol HP, and glycerol, based on their respective HLB values and ionic properties [18]. A surplus amount of terbinafine HCL (TH), which is the drug, was introduced to 2 mL of the excipient (either oil or surfactant) in an Eppendorf tube. This mixture was then vigorously mixed using a Remi CM-101 cyclomixer for 72 h at a temperature of 25 °C. Subsequently, it underwent centrifugation in a Remi R8C laboratory centrifuge at a speed of 3000 rpm for 10 min, allowing any excess drug to settle. The supernatant liquid was collected, diluted with methanol, and filtered through a 0.22 μm syringe filter. The resulting diluted samples were analyzed using a UV spectrophotometer at a wavelength of λmax 283 nm to determine the concentration of the solubilized drug.

#### 4.2.2. Pseudo-Phase Ternary Diagrams

The choice of oil, surfactant, and co-surfactant was determined based on their ability to maximize the solubility of the drug within these ingredients. To create the nanoemulsion, we employed the aqueous microtitration method. Initially, we assessed pre-selected oils in combination with a surfactant and co-surfactant mixture (referred to as S_mix_). This evaluation involved mixing the oil and S_mix_ in various ratios to create a clear solution, followed by dilution with distilled water [19]. This step was crucial for assessing the compatibility and miscibility of the oils with S_mix_, with different ratios such as 1:1, 2:1, 3:1, 1:3, and 1:2 being tested. 

Additionally, we conducted aqueous dilution steps to visually observe clarity and turbidity after each dilution, ultimately leading to the formation of a clear nanoemulsion. Furthermore, we explored various oil-to-S_mix_ ratios, including 1:9, 1:8, and 1:4, to expand the nanoemulsion region and confirm the formulation of an oil-in-water (o/w) nanoemulsion. To map out the composition space, we constructed pseudo-ternary phase diagrams using (PCP-Triangular software).

#### 4.2.3. Formulation of Terbinafine HCl Nanoemulsion (TH-NE)

The nanoemulsion described earlier was created through a self-emulsification process. Initially, 0.625 mg of TH was dissolved in 120 µL of olive oil and then mixed with a solubilizing agent, S_mix_ (consisting of propylene glycol and Span 80 in a 1:2 ratio). Afterward, this pre-emulsion underwent vortex mixing for 10 min, resulting in the formation of a nanoscale formulation.

#### 4.2.4. Thermodynamic Stability Studies

Thermodynamic stability testing was performed in order to mitigate the potential emergence of an unstable formulation. The experimental procedure involved testing the formulation through various methods. This included subjecting it to centrifugation at 3000 rpm for 20 min, as well as exposing it to alternating cycles of heating and cooling between temperatures of 4 and 45 °C for 24 h. Additionally, a freeze–thaw cycle was carried out by storing the formulation at −21 °C for 24 h, followed by storage at +21 °C. The nanoemulsion was visually inspected to detect any signs of instability [20].

#### 4.2.5. Optimization of Terbinafine HCL-Nanoemulsion (TH-NE) Using Design of Expert (DoE)

The optimization of various TH-NE formulations was carried out using DoE software (version 132.0.4, Stat-Ease, Minneapolis, MN, USA). This involved the consideration of both independent and dependent variables. The chosen independent variables included stirring speed (rpm), surfactant concentration (mL), and oil concentration (µL), while the dependent variables consisted of particle size (nm), PDI, and zeta potential (mV). Table 4 displays the designated high, medium, and low levels for oil and surfactant concentrations, as well as stirring speed, as specified in the DoE process. The optimization procedure adhered to the Box–Behnken design (BBD), a response surface methodology employed to predict how changes in independent variables impact dependent responses [15]. A total of 15 randomized experimental runs were conducted, and the data were analyzed using the BBD to ultimately obtain an optimized formulation.

#### 4.2.6. Particle Size, Polydispersity Index (PDI), and Zeta Potential 

Dynamic light scattering (DLS; Malvern Zetasizer, Nano ZS, Worcestershire, UK) was used to measure the particle size, PDI, and zeta potential of the optimized TH-NE. Prior to and throughout the analysis, the following conditions were kept constant: the formula was diluted 1:50, the temperature was held steady at 25 °C, and the scattering angle was set to 90 degrees. 

#### 4.2.7. Differential Scanning Calorimetry (DSC) of the Lyophilized Formulation

The Pyris 6 DSC, Perkin Elmer, Waltham, MA, USA, was used for the DSC analysis of both TH-NE and the lyophilized formulation of TH-NE. Each sample went through a quick procedure. We took a 2 mg sample and heated it from 25 to 350 °C. Nitrogen flow was kept constant at 60 mL/min, while the temperature was raised at 10 °C/min.

#### 4.2.8. Fluorescent Microscopy

A fluorescence microscopy examination was carried out so that the morphology of the TH-NE could be determined. The improved TH-NE mixture was diluted 10 times with milli-Q water and further treated with rhodamine B, a water-soluble fluorescent dye, before being observed under a fluorescence microscope [21]. In brief, 2 mL of TH-NE was labeled with rhodamine dye, and the mixture was left alone for 10 min. In addition, the slides were prepared with the complex that included rhodamine, and the TH-NE was imaged afterward.

#### 4.2.9. Preparation of Drug-Loaded Nanoemulgel Using Optimized Terbinafine HCl Nanoemulsion (TH-NEG)

The improved TH-NEG formulation was developed as an emulgel using different ratios of the gelling component, Carbopol 934^®^, at a concentration of 1% (*w*/*v*). The procedure involved first dispersing Carbopol 934^®^ in distilled water, followed by adding TH-NE (5 mL) while stirring continuously. To ensure a uniform nanoemulgel dispersion and remove any air bubbles, the resulting mixture was sonicated for 15 min.

#### 4.2.10. Rheological Behavior 

The rheological properties of the gels were assessed utilizing a controlled stress rheometer, namely the Physica MCR 101 Anton Parr Rheometer. Both gels were tested to evaluate their mechanical properties using oscillating and flow tests [22]. The rheometer produced mechanical spectra across a frequency range of 0.01 to 10 Hz. Stress sweep experiments were conducted at a consistent frequency of 1 Hz to ascertain the viscoelastic properties of the materials under investigation. The experiments were carried out in a controlled environment at a temperature of 25 ± 1 °C, using spindle number PP50 and the cone-and-plate technique [23].

#### 4.2.11. Spreadability

A glass plate was utilized as the substrate for a TH-NEG sample weighing 0.5 g. The glass plate was initially inscribed with a circle measuring 1 cm in diameter. A second glass plate, with a mass of 500 g, was placed on the top of the sample for a period of 5 min. The expansion of the gel’s surface diameter, in this case, reflects its spreading capacity, and you can determine it using the provided formula:

W × L/t = S

Where S stands for spreadability (measured in grams per second), W represents the applied weight on the glass plate (measured in grams), L signifies the distance the glass plate moves, and t denotes the elapsed time [24].

The experiment was conducted in triplicate, employing a linear scale to measure the extension necessary for separating the glass plates.

#### 4.2.12. Extrudability

Extrudability refers to the degree of ease with which TH-NEG can be expelled from a tube, specifically indicating the distance of 0.5 cm that the formulation can be expelled within 10s. Firm pressure was applied to the crimped end of a closed collapsible tube containing a pre-weighed amount of the optimized TH-NEG. After a set period, the pressure was relieved, and the tube’s cap was removed to measure the amount of gel that had been pushed out using a linear scale. There exists a direct relationship between the amount of extruded gel and the ease of extrusion [17].

#### 4.2.13. Texture Analyzer

The TH-NEG sample was positioned on a level testing surface within a glass jar to prevent premature activation and the formation of air bubbles. The evaluation of the firmness, cohesiveness, consistency, and viscosity of the sample was performed using a texture analyzer (TA. Plus, Stable Micro Systems, Surrey, UK) in a compression mode [10]. Force–time graphs were utilized to assess various texture profile attributes of TH-NEG.

#### 4.2.14. Homogeneity and pH

A visual inspection was utilized to evaluate the consistency of the optimized TH-NEG within a transparent glass vessel [25]. The pH measurement was conducted using a digital pH meter (HI 98107, Hanna Instruments, Navi Mumba, India) equipped with a glass microelectrode. The temperature was carefully controlled at 25 ± 1 °C, and a 1 min equilibration period was observed before taking the measurements.

#### 4.2.15. In Vitro Drug Release Study

The release profiles of terbinafine hydrochloride (TH) in the TH-NEG, TH-NE, and marketed formulations of terbinafine hydrochloride cream IP (specifically Taxifen^®^ cream containing 5 mg terbinafine HCL) were evaluated using a pre-activated dialysis bag with a molecular weight of 12,000 Dalton [26]. The dissolution media employed in this study consisted of a 250 mL volume of phosphate buffer solution with a pH of 5.5. The dissolution process was carried out at a rotational speed of 100 revolutions per min and a controlled temperature of 37 ± 0.5 °C. Subsequently, 2 mL aliquots were obtained at predetermined time intervals (5 min, 10 min, 15 min, 30 min, 1 h, 2 h, 4 h, 6 h, 12 h, 24 h, 36 h, and 48 h) and promptly substituted with an equivalent volume of phosphate buffer to maintain sink conditions. The collected sample was subsequently subjected to filtration, dilution, and analysis utilizing UV spectroscopy at a specific wavelength of 282.50 nm.

#### 4.2.16. Ex Vivo Release Study

Hairless mice skin excised from the abdominal region is utilized for ex vivo studies. The surgical procedure involved the use of isopropyl alcohol to eliminate subcutaneous fat [2]. The hairless and prepared mice skin was introduced into a Franz diffusion cell in order to evaluate the permeation behavior of the TH-NEG, TH-NE, and commercially available formulation within the donor compartment, which contained 2 mL of the sample. In the receptor compartment, a volume of 10 mL of phosphate-buffered saline (PBS) with a pH of 5.5 was introduced as the dissolution media. The temperature was maintained at 37 ± 0.5 °C, and a constant stirring speed of 100 rpm was applied. The aliquot (2 mL) was collected and replaced with a new and equal volume of dissolving medium at predetermined time intervals (1, 2, 4, 6, and 12 h) [27]. The obtained aliquot was subjected to additional filtration, dilution, and subsequent analysis utilizing UV spectroscopy at a wavelength of 282.50 nm.

#### 4.2.17. Confocal Laser Scanning Microscopy

The present study employed murine skin samples that were prepared according to the methods described in the literature. The skin tissues were positioned within the donor and receptor chambers of the Franz diffusion cell in order to evaluate the tissue uptake. In the experiment, a rhodamine B aqueous solution with a concentration of 0.03% weight/volume was employed in the donor compartments. Additionally, TH-NEG and TH-NE with rhodamine were also utilized in the same compartments. Conversely, the receiver compartments were filled with a phosphate buffer solution at a pH of 5.5 [28]. Following 24 h, the skin samples were procured and subjected to a thorough cleansing process involving the use of distilled water and alcohol. The skin samples were subsequently positioned on slides in such a way that the stratum corneum, which is a layer of the skin, faced upward. These samples were then labeled using an argon laser beam with an excitation wavelength of 540 nm and an emission wavelength of 625 nm. This labeling process was carried out using a fluorescence microscope known as the Olympus FluoView TM FV1000, which is manufactured in Hamburg, Germany. 

#### 4.2.18. Dermatokinetic Study 

The dermatokinetic analysis involved studying drug concentrations in different skin layers. Skin samples were collected at various time points (0, 0.5, 1, 1.5, 2, 3, 4, 5, 6, and 8 h) from the diffusion cell [29]. To remove any remaining formulation on the skin, normal saline was used for skin washing. Subsequently, the skin was briefly immersed in heated water (60 °C) for 2–3 min. The epidermal and dermal layers were then carefully separated using blunt and fine forceps. These isolated layers were finely minced and soaked in 5 mL of methanol to extract the drugs. Afterward, a 0.22 μm pore size filter was utilized to filter the methanolic solution, and the drug quantity in the solution was determined using HPLC. All measurements were conducted in triplicate. The concentration of TH per cm^2^ of skin was plotted over time for both the epidermis and dermis. Dermatokinetic parameters such as T_skin max_, C_skin max_, AUC_0–8 h_, and Ke were determined using PK solver software.

#### 4.2.19. Statistical Analysis

All data are presented as the mean ± standard deviation (SD) of six replicates. GraphPad Prism software was utilized for the statistical analysis (Instat 3.06, San Diego, CA, USA). Comparisons between the two samples were performed using Student’s t-test. For statistical analysis of various parameters in the pharmacokinetic part, PK Solver 1.0 (Microsoft Corp., Redmond, WA, USA) was employed. *p*-values < 0.05 were regarded to be significant.

## Figures and Tables

**Table 1:** Responses of the dependent factors such as particle size, PDI and zeta potential.

S.NO.	Factor 1 A: Surfactant Concentration (mL)	Factor 2 B: Oil Concentration (µL)	Factor 3 C: Stirring Speed (rpm)	Response 1 Particle Size (nm)	Response 2 PDI	Response 3 Zeta Potential (mV)
1	1.5	0.16	150	28.09	0.1911	−40.9
2	1	0.12	150	28.21	0.182	−40.3
3	1	0.16	100	28.6	0.191	−40.2
4	1	0.12	150	28.12	0.195	−40.5
5	1	0.12	150	28.07	0.1922	−41.87
6	0.5	0.12	100	31.2	0.298	−38.9
7	1	0.08	200	27.9	0.189	−37.02
8	1.5	0.12	200	27.25	0.184	−37.07
9	1	0.16	200	31.01	0.295	−39.9
10	1.5	0.08	150	28.9	0.211	−39.7
11	0.5	0.16	150	32.04	0.302	−38.7
12	1.5	0.12	100	28.97	0.219	−39.5
13	1	0.08	100	31	0.28	−38.65
14	0.5	0.08	150	29	0.215	−39.6
15	0.5	0.12	200	29	0.227	−39.5

**Table 2:** Regression analysis summary of independent variables on responses R1 (particle size), R2 (PDI), and R3 (zeta potential).

Responses	R^2^	Adjusted R^2^	Predicted R^2^	S.D.	C.V.%	Adequate Precision
R1 = Particle Size	0.9452	0.8467	0.1286	0.5663	1.94	9.8076
R2 = PDI	0.9237	0.7864	−0.1757	0.0209	9.30	7.5459
R3 = Zeta Potential	0.7583	0.3233	−2.0193	1.07	2.71	3.8354

**Table 3:** Data from texture analysis showing the measurements for TH-NEG, including parameters such as firmness, consistency, cohesiveness, and cohesion.

Firmness [g] Force 1	Consistency [g/s] Area F-T 1:2p	Cohesiveness [g] Force 2	Work of Cohesion [g/s] Area F-T 2:3
168.00	229.81	−83.36	−107.02

**Table 4:** Variables and their constraints in Box–Behnken design.

Variables	Constraints		
	Lower Limit	Upper Limit	Middle Limit
Independent variables			
Stirring speed (rpm)	100	200	150
Surfactant concentration (mL)	0.5	1.5	1
Oil concentration (µL)	0.08	0.16	0.12
Dependent variables			
Particle size (nm)	Minimum		
PDI	Minimum		
Zeta potential (mV)	−40 to +40		

## Data Availability

The data presented in this study are available in this article.
